# Risk Factors Associated With Birth Asphyxia in Term Newborns at a Tertiary Care Hospital of Multan, Pakistan

**DOI:** 10.7759/cureus.18759

**Published:** 2021-10-13

**Authors:** Ghazanfar Nadeem, Abdul Rehman, Humaira Bashir

**Affiliations:** 1 Department of Neonatology, The Children's Hospital & Institute of Child Health, Multan, PAK; 2 Department of Neonatology, The Children’s Hospital & Institute of Child Health, Multan, PAK; 3 Department of Obstetrics & Gynecology, Nishtar Hospital & Medical University, Multan, PAK

**Keywords:** risk factors, newborn, asphyxia, apgar score, amniotic fluid

## Abstract

Background

Perinatal asphyxia is one of the main causes of death in term newborns. During the past two decades, no significant progress has been made in reducing neonatal deaths in developing countries. This study was aimed to determine various factors associated with birth asphyxia in term newborns at a tertiary care hospital of Multan, Pakistan.

Methods

This case-control study was conducted at the Neonatal pediatrics Department, The Children’s Hospital, Multan in collaboration with the labor room of Nishtar Hospital Multan from April 2020 to September 2020. Newborns delivered in the labor room with a low Apgar score of five or less at the first minute were recruited as cases and newborns with an Apgar score of more than five in the first minute were recruited as controls. The demographic information of neonates and mothers was noted. A Performa was used to collect all information. All data were analyzed through SPSS 26.0 (IBM SPSS Inc., Chicago, IL, USA).

Results

A total of 426 newborns (213 cases and 213 controls) were enrolled. In cases, there were 132 males and 81 females whereas there were 115 males and 98 females in the control group (P=0.09). Majority 132 (62%) mothers of cases were primiparous compared with 110 (52%) mothers of control. The difference in parity of mothers of cases and control (P=0.03) was significant. Prolonged labour was noted in cases 123 (58%) vs. controls 55 (26%) (P=0.001) while fetal distress was found in 120 (56%) cases and 45 (21%) controls (P=0.001). Meconium was found in the amniotic fluid in 171 (80%) cases and 86 (40%) controls (P=0.001). All other risk factors turned out to be insignificant between cases and controls (P>0.05).

Conclusion

Meconium stained liquor is a major risk factor for birth asphyxia. Prolonged labor of more than 24-hour period, as well as fetal distress, is also a major risk factor of perinatal asphyxia. Involving obstetricians in the present research give more reliability and reproducibility of the data collected.

## Introduction

Perinatal asphyxia is one of the main causes of death in term newborns [1]. During the past two decades, no significant progress has been made in reducing neonatal deaths in developing countries. According to World Health Organization (WHO) birth asphyxia is a failure to initiate and sustain breathing at birth [2]. lack of effective respiration results in hypoxemia (lack of oxygen) and hypercapnia (accumulation of carbon dioxide) [3], both of these depress cardiac function. In asphyxiated newborns, severe hypoxic damage occurs in many organs, including the brain, heart, kidney, liver, lungs, and gut but brain damage is of most concern and perhaps the least likely to recover [4]. Birth asphyxia is a serious medical problem worldwide, especially in developing countries, and the main contributor to neonatal mortality and morbidity [3].

Risk factors of birth asphyxia have been divided into antepartum and Intrapartum factors. Antepartum factors include high or low maternal age, Primigravida or grand multipara birth, and eclampsia. Intrapartum factors are meconium-stained liquor, fetal distress, and prolonged labor [5,6].

The objective of this study was to detect various factors responsible for asphyxia in the term newborns. Recognition of these factors before birth and early commencement of preventive measures can reduce neonatal mortality and brain injury due to birth asphyxia.

## Materials and methods

This case-control study was conducted at the Department of Neonatology, The Children’s Hospital, Multan, Pakistan, in collaboration with the labor room of Nishtar Hospital Multan. Approval from The Ethical Committee of The Children's Hospital & Institute of Child Health, Multan, Pakistan, references number ECP/2021/249 was acquired. Written and informed consent was taken from all mothers. The data was collected during the six-month period from April to September 2020. Mothers of newborn pairs who were delivered in the labor room were included in the study.

A total of 426 newborns (213 cases and 213 controls) with gestational age more than or equal to 37 weeks to 42 weeks and delivering a single live-birth weighing between 2,000 and 4,000 grams were recruited. Babies with a low Apgar score of five or less at the first minute after birth were recruited as cases and babies with an Apgar score of more than five at first minute after birth were recruited as control. Apgar score at one minute after birth was calculated according to the standard criteria by attending neonatal physicians in the labor room. All mothers with gestational age less than 37 weeks were excluded.

EPI INFO was used to calculate sample size according to double population formula considering the proportion of Primigravida in case group 59% and control group 45% from the previous study conducted in Nigeria [7]. By considering 95% CI and 80% power, the total sample size turned out to be 426 (213 cases and 213 controls).

A special proforma was used to collect study data. The basic demographic information included the age of the mother, gender of baby, birth weight, and mode of delivery. Dubowitz method was used to determine the gestational age of all the newborns [8]. Information about the four selected risk factors of birth asphyxia i.e parity, fetal distress, prolonged labor, and meconium-stained liquor was obtained from the gynecologist attending the delivery in the labor room. Study variables were Primigravida, fetal distress, prolonged labor, and meconium-stained liquor. In all newborns, the time span of labor was assessed as the time interval between the onset of regular uterine contractions to the expulsion of the baby. Prolonged labor was taken as labor lasting over 24 hours.

All the data were analyzed through SPSS version 26.0 (IBM SPSS Inc., Chicago, IL, USA). Mean and standard deviation were calculated for the age of the mother, gestational age, and weight of the baby. Frequency and percentages were calculated for the gender of the babies and mode of delivery. Confounding factors like the age of the mother, weight of the baby, and gestational age of the baby were controlled by stratification. A Chi-square test was applied to compare the occurrence of study variables (Primigravida, fetal distress, prolonged labor, and meconium-stained liquor) in cases and controls. P-value equal to or less than 0.05 was taken as significant.

## Results

The majority of the newborns, 247 (58.0%) were male. The mean birth weight of the cases was 2,823.47+478.37 grams whereas the mean birth weight of control was 2,848.36+438.26 grams. There were 252 (59.2%) mothers who had a mode of delivery as spontaneous vaginal birth while the remaining 174 (40.8%) delivered by cesarean section. The mean age of the mothers in the case group was 26.35­+6.79 years range (15-42 years) while the mean age of controls was 25.76+6.46 (15-42 years). Table 1 is showing the comparison of baseline characteristics of cases and controls where no statistically significant difference was noted between the two study groups (P>0.05). The mean Apgar score in the case group was 4.093+1.06 while it was noted to be 7.906+1.18 among controls.

**Table 1:** Baseline characteristics in cases and controls (n=426)

Characteristics		Cases (n=213)	Controls (n=213)	P-value
Gender	Male	132 (62.0%)	115 (54.0%)	0.0952
	Female	81 (38.0%)	98 (46.0%)	
Birth Weight (grams)	2,000-2,400	52 (24.4%)	38 (17.8%)	0.1181
	2,500-3,500	145 (68.1%)	164 (77.0%)	
	3,600-4,000	16 (7.5%)	11 (5.2%)	
Mode of Delivery	Spontaneous Vaginal Delivery	117 (54.9%)	135 (63.4%)	0.0760
	Cesarean Section	96 (45.1%)	78 (36.6%)	
Age of Mothers (years)	< 18	23 (10.8%)	18 (8.5%)	0.4874
	18-35	164 (77.0%)	174 (81.7%)	
	>35	26 (12.2%)	21 (9.8%)	
Gestational Age	37-38 weeks	100 (46.9%)	119 (55.9%)	0.1542
	39-40 weeks	94 (44.1%)	81 (38.0%)	
	>40 weeks	19 (9.0%)	13 (6.1%)	

Table 2 compares the study variables in cases and controls. The majority 132 (62.0%) of mothers of cases were Primigravida compared with 110 (51.6%) of mothers of control. There was a significant difference in parities of mothers of cases and control (P=0.0314). Fetal distress was found in 120 (56.3%) cases and 45 (21.1%) of control (P<0.0001). Meconium was found in the amniotic fluid in 171 (80.3%) cases and 86 (40.4%) of the controls (P<0.0001). Prolonged labor was noted in 123 (57.7%) cases in comparison to 55 (25.8%) controls (P<0.0001).

**Table 2:** Comparison of study variables in cases and controls (n=426)

Risk Factor		Case (n=213)	Control (n=213)	P-value
Parity	Primigravida	132 (62.0%)	110 (51.6%)	0.0314
	Multigravida	81 (38.0%)	103 (48.4%)	
Fetal Distress	Yes	120 (56.3%)	45 (21.1%)	<0.0001
	No	93 (43.7%)	168 (78.9%)	
Meconium in Amniotic Fluid	Meconium Stained	171 (80.3%)	86 (40.4%)	<0.0001
	No Meconium	42 (19.7%)	127 (59.6%)	
Prolonged labour	Yes	123 (57.7%)	55 (25.8%)	<0.0001
	No	90 (42.3%)	158 (74.2%)	

## Discussion

This study was conducted to identify the various factors leading to perinatal asphyxia in term newborns delivered at Nishtar Hospital Multan, Pakistan. In the present study, 62.0% of cases were male in comparison to 54.0% controls but the difference was not statistically significant. A study from the Children Hospital Lahore, Pakistan, found a male to female ratio of 2:6:1 in newborns with asphyxia [9]. Our study showed Primigravida as an important risk factor of birth asphyxia (62% cases vs. 51.6% controls, P=0.0314). This is in collaboration with studies conducted in India and Nepal [10,11].

The Primigravida are often being deprived of the demands of pregnancy like early booking and regular antenatal visit therefore they are unaware of their responsibility to themselves and their unborn fetus [12]. This may cause complications that lead to birth asphyxia. Fetal distress was also found to be a significant factor between cases and controls in the present study (56.3% vs. 21.1%, P<0.0001). Studies showing similar findings have been published in the past [13,14].

Meconium-stained liquor was seen in 80.3% cases in comparison to 40.4% controls (P<0.0001). The presence of meconium provides an additional criterion for the determination of fetal distress. Grade III or grade IV meconium staining is considered to be a maker of more prolonged or severe asphyxia episodes [3]. So, meconium in liquor is another risk factor of birth asphyxia. This was according to studies conducted in India, Nigeria, Yaounde Tertiary Hospital Cameron and University of Gondar Referral Hospital [7,8,15,16]. Prolonged labor was also observed as a significant risk factor for birth asphyxia (P<0.0001). This was similar to the studies conducted in the past in different parts of the world like Dhaka medical college hospital, Jimma Medical Center Ethiopia, Referral Hospitals of Amhara National Regional State Ethiopia, Nigeria, and from Stockholm and Gotland Sweden [17-21].

Although we did not find any significant association of gestational age with the risk of birth asphyxia, some studies have shown increasing trend of birth asphyxia with an increase in gestational age [1,22]. Our study also showed that birth asphyxia is more common between 18 and 35 years of age of the mother although it did not reach statistical significance. More newborn in cases were delivered by cesarean section in comparison to controls but the difference did not reach statistical significance (45.1% vs. 36.6%, P=0.0760). Some researchers have found emergency cesarean section due to any reason to be an important risk factor of asphyxia [7,23,24].

Being a case-control study is one of the strengths of this research. There were some limitations in our study as this was a single-center study and findings cannot be generalized. We were unable to collect information regarding maternal diabetes, hypertension, or the presence of anemia, which could have given us further insight. We could not evaluate cord blood gases in diagnosing perinatal asphyxia. Moreover, Apgar scores at 2, 5, and 10 minutes would have further given us very useful information.

## Conclusions

Meconium-stained liquor is a major risk factor for birth asphyxia. Prolonged labor of more than 24-hour period, as well as fetal distress, is also a major risk factor of perinatal asphyxia.

Involving obstetricians in the present research give more reliability and reproducibility of the data collected. The incidence of perinatal asphyxia in any community is too large magnitude dependent on main risk factors; these, in turn, being influenced by the effect of literacy level, ethnic and customary views, health education, and resourceful application of health care facilities as well as the quality of obstetric, gynecological, and newborn care.
